# Practical aspects of laboratory automation in pharmaceutical development

**DOI:** 10.1155/S1463924693000021

**Published:** 1993

**Authors:** Eugene J. McGonigle

**Affiliations:** Physical & Analytical Chemistry Research & Development Schering Plough Research Institute Kenilworth New Jersey USA


Journal of Automatic Chemistry, Vol. 15, No. 1 (January–February 1993), pp. 3–8

### Practical aspects of laboratory automation in pharmaceutical development

**Eugene J. McGonigle**
*Physical & Analytical Chemistry, Research & Development, Schering Plough Research Institute, Kenilworth, New Jersey, USA*

### Introduction

In the pharmaceutical industry the most exciting aspect of Research & Development is the discovery of a new chemical entity with one or more unique therapeutic properties that are generally recognized and accepted as an advance in safe and effective treatment of disease. The discovery and transformation of this compound into a product which submitted to the authorities can take up to 10 years, costing millions of dollars.

It is the mission of the pharmaceutical development divisions to provide a stable dosage form(s) for evaluation in clinical trials and, ultimately, a competitive market image which can be manufactured at large scale, with reproducible purity and quality and a shelf-life consistent with worldwide environmental conditions. Therefore, analytical testing in R&D laboratories, using stability indicating methods, plays a pivotal role in many of the decisions which lead to the ultimate definition of 'a final product'.

The most time-consuming analyses that occur in analytical R&D laboratories are those associated with stability testing. The minimum requirement is that three batches of the product be manufactured, representative of the commercial manufacturing process, equipment and scale. These are then packaged in each type and size of container which will be used in the market-place. For example, for a tablet dosage form, this might be glass and polyethylene bottles and blisters. All of these are then placed at stability stations, simulating worldwide environmental limits to which the product will be exposed and analytically tested at fixed intervals over a period of up to three to five years. In addition, the samples are subjected to additional testing at conditions which are more stressful with the hope that one might project, kinetically, changes which could highlight a stability problem early in the stability protocol. A typical stability protocol for a single dose of a solid dosage form translates into 648 determinations in the first year exclusive of analytical reference standards which must be used in calculations. When this is multiplied by the number of dosage strengths for which a product is developed, a single stability protocol can easily effect close to 2000 determinations in the first year of testing. In 1991 the author's Analytical Reseach & Development Laboratories generated more than 225 000 analytical determinations, utilizing the efforts of 89 bench chemists. This translated into approximately 11·5 completed deteminations per chemist/day. With numbers of this magnitude, one can appreciate the role for automation and laboratory efficiency with minimal chance for error (see figures 1 and 2).

This paper was read at the International Symposium on Laboratory Automation and Robotics (October 1992, Boston, USA).

### Laboratory automation

Laboratory automation *is* 'Efficiency', *is* 'Computers'. Prior to the 1970s, chromatographic methods were conducted manually. Generally, procedures consisted of: weighings, extractions, dilutions, filtering and/or centrifugation, injections and manually determined measurements of chromatographic peak heights and calculations. All raw data were hand-transcribed into

Three batches
Representative of commercial process/scale/equipment
Packaged:
All types/sizes of marketed containers
Stability stations:
- 25 and 30 °C/60% RH
- Refrigeration

Accelerated conditions:
- Light (500 ft Cand)
- 40 °C/80% RH
- 50 °C

Testing intervals:
- Three month intervals/first year
- Six month intervals/thereafter

Analytical testing:
- Stability indicating assay
- Content uniformity (initial only)
- Dissolution (solid dosage forms)
- Decomposition products

*Figure 1. General stability requirements.*

Three batches/three packages = nine samples
Initial assay/three months/six months/12months = five intervals
Four tests conducted intially/three thereafter
Taken into account: complete testing not conducted at every condition
*Result:* 324 samples tested
648 determinations conducted (duplicate testing)
*Note:* Standards not included

*Figure 2. Typical stability protocol for one product/one dose: number of determinations.*

© Zymark Corporation 1993

notebooks, along with strip chart recordings. For a typical gas chromatographic analysis of a two-component, tablet dosage form, a chemist might complete five samples per day. In the 1970s, efficient autoinjectors and autosamplers came on the scene, together with early versions of 'LAS' (Laboratory Automation Systems). These inroads effected computerized area integration and calculations, while providing hard-copy records of calculated results of laboratory notebooks. With these innovations, a chemist could now complete in the region of 10 samples in one and one half days. Robotics were added to the laboratory 'arsenal', in the 1980s which freed the chemist from many time consuming manipulations and allowed these operations to be carried out 'off-hours'. LAS systems were more sophisticated, allowing baseline assessment and more reliable raw data files for records and audits. Today, it is possible for a chemist to generate twice the workload in half the time required in the past (See figures 3–5).

In the author's research analytical laboratory, the opportunities to be innovative in automation require a careful assessment of the principle day-to-day laboratory operations. These can change from time to time. While integrated systems are the ultimate goal, it has been found that, even if considered separately, operations can be automated and made more efficient using 'distinct systems', rather then 'force' a premature integration of systems that appear to do everything. In Schering-Plough's laboratories, the principle operations have been segregated into various categories:

(1) Logging/scheduling of samples.
(2) Analysis: manipulations; instrumental analysis; calculations.
(3) Data handling to effect formatted results.
(4) Tabulated presentations/reports/statistics.
(5) Productivity measurements: number of determinations; project accountability; headcount justification.

The following components meet most of the author's laboratory automation needs:

(*a*) Autosamplers: direct measurements, chromatographic injections, dilutions, etc.
(*b*) Robotic systems: manipulations.
(*c*) Laboratory automation systems: chromatographic integrations; calculations; raw/processed data files.
(*d*) Database management systems: LAS Interface; stability data files; software to 'act on' the database.
(*e*) Spread sheet: productivity assessment.

The payback for these is obvious. *Scheduling* is simplified as is a readily available archive. One can more effectively monitor and control sample backlog. Compliance issues can be rapidly addressed regarding sample identification and disposition among various laboratories, chemists and notebooks. *Analysis* may be conducted with minimal operator manipulations. Not only does this maximize sample turnover, but it also minimizes the chance for errors. *Data handling* is streamlined, with interim data reports and trend analyses being carried out in a fraction of the time formerly required. Regulatory quality reports and tablets can be rapidly generated with minimal transcription required. Finally, *productivity measurements* can be generated on a monthly basis, if desired, which trend laboratory workload, productivity and resource needs and availability.

However, these paybacks are not without a price. These systems require extensive *validation/re-validation* and specific inspection criteria versus regulatory guidelines, which constantly evolve. *Additional calibration* and *preventive maintenance* are necessary, since malfunctions can be subtle. General use in the laboratories requires additional *department SOPs and consensus agreement* among supervisors, which can create individual manager restrictions and boundaries which are less than popular. Further, many analyses need to be *customized* to facilitate automation. Time studies and project loads need to be assessed, for example is it more productive to limit automation to sample preparation or should chromatographic injections be included? Because these systems are in daily use, they become taken for granted, leading to the potential for

**Pre 1970s**

- Manual weighing
- Manual liquid/liquid and/or liquid/solid extraction
- Manual dilution
- Manual centrifugation
- Manual syringe injection
- Manual peak height calculations
- Raw data manually recorded in notebooks

Result: one chemist – five samples per day

*Figure 3. Evolution of a typical gas chromatographic assay: two components in a single tablet.*

**1970s**

- Manual weighing
- Manual liquid/liquid and/or liquid/solid extraction
- Manual dilution
- Manual centrifugation
- Automated injectors
- Integration of areas (early versions of LAS)
- Printed data files for notebook entry

Result: one chemist – 10 samples in 1½ days

*Figure 4. Evolution of a typical gas chromatographic assay: two components in a single tablet.*

**1980s**

Robotics: weighing; extractions; dilutions; centrifugation
Automated injectors
Integration of areas (advanced LAS)/reliable raw data files
Printed data files for notebook entry

Result: One Chemist – 30–40 samples per 1½ days

*Figure 5. Evolution of a typical gas chromatographic assay: two components in a single tablet.*

significant impact on laboratory productivity in the event of *computer malfunction* or *system failure*. Finally, one must guard against the impression that *analyses are completed* when, in reality, *results* are unavailable due to the chemist waiting for extensive print-outs and completion of paperwork.

While these tradeoffs have to be considered, there can be little doubt that automated systems benefit most laboratories when assessed with perspective versus day-to-day needs. The use of three systems in the author's Analytical Research and Development Laboratories is discussed, and the qualitative and quantitative conclusions that have been drawn as to their contribution to the daily operations are given. The systems are: LAS, robotics and spreadsheet analysis. Overall, it is important to remember that payback can always be looked at in quantitative terms. Increased productivity can result for better audit trails and better guards against error, although the effort can require more input. Every laboratory is sufficiently unique to the extent that their experiences are often different. Therefore, in some respects, the following discussion is obvious with regard to specific brands of equipment, it has been limited to the author's experience with systems and not specific brands.

### Laboratory Automation System (LAS)

The author's view of an LAS system is that it facilitates chromatographic injections/integrations, calculations and data presentations. From a regulatory point of view, it reduces the subjectivity that the chemist adds to the review of chromatographic baselines, and subsequent decisions which he or she must make regarding various integration parameters. The system will present the area for integration versus proposed parameters, allowing the chemist to take into account subtle, regional aberrations in the tracing through the ability to amplify any specified region of the chromatogram through scale expansion. More importantly, the supervisor can review sample tracings to verify conclusions drawn by the chemist. The result is that integrations are both rapid and normalized versus fixed criteria. Calculations can be rationalized. New peaks, which might signify impurities and/or degradation products, can be assessed and quantified. Processed data files provide untranscribed records of sample identification, calculated results and integration parameters. Raw data files can be retained for review and verification at some future date. From a practical point of view, one cannot dispute the significance of the above, regulatory advantages. The advantages require operator input, but ensure quality data, an important but more intangible contribution to productivity. To determine a quantitative net effect, the time required to manually complete a series of calculated results was monitored, for a typical HPLC chromatographic analysis, and compared to that required using the LAS system. The protocol consisted of an HPLC analysis of 18 samples of a dosage form containing an enantiomeric compound. The comparison did not include sample manipulations including chromatographic injections. We found the overall time savings remains substantial despite the more detailed review the chemist was afforded using LAS. The assessment is as shown in table 1.

From the point of view of elapsed analysis time, utilization of LAS/Data Analysis does not affect this significantly since analysis time is chromatography-driven (LAS amasses data with each chromatogram). However, LAS allows analyses to continue off-hours. Therefore, up to three times the number of samples can be processed. A major advantage of all forms of automation is the conduct of analysis off-hours. In the author's laboratories, 70–80% of all chromatographic analyses are conducted in this fashion with the chemist, ideally, devoting an eight hour work day to prepare samples which require up to 16 hours of chromatography time. While this may seem self-evident, one has to consider that were the sample load limited to, say, five samples (10 determinations) it may not be worth overloading a fully utilized LAS system for this work since these analyses could possibly be efficiently completed manually by a chemist in a single working day. Were there a series of such situations occurring simultaneously, utilization of LAS could actually decrease productivity (by slowing LAS response), with only the regulatory advantages remaining the pivotal consideration. Power system failures used to be a major limitation to LAS systems but constant power sources have virtually eliminated these problems and this equipment is really no more limited than any other laboratory instrument. Computer malfunction, or system failure, is potentially a far more serious situation and must be weighed into the commitment a laboratory makes regarding all laboratory automation, particularly if integrated systems are used.

### Robotics

While LAS has contributed to efficiency in chromatographic analyses, robotics has significantly reduced operator time in carrying out sample preparation. Early versions of robotics (pre 1980s) were limited for the most part to quality control laboratories dealing with high-volume marketed products. The rigid/fixed configurations of early robotic systems were not readily amenable to the changeover required for different products. In addition, method development required several months, in part, related to robotic programming. Highly trained staff were necessary for dedicated commitment to utilizing the robot.

In R&D, two attributes in the nature of the workload play a pivotal role determining the practicality of an invest-

*Table 1. LAS compared with manual procedure.*

| Activity | Operator manually | Operator LAS |
|---|---|---|
| Define/Draw baselines | 1·7 h | 0·5 h |
| Measure peak heights | 1·7 h | 0·0 h |
| Tabulate values | 0·5 h | 0·0 h |
| Calculations | 3·0 h | 0·0 h |
| Notebook completion | 0·5 h | 0·5 h |
| *Total* | 7·4 h | 1·0 h |

ment in a robotic system. First, R&D projects evolve in stages. Early in clinical trials, preliminary formulations/ doses are used. As the clincial program progresses, additional doses are identified as are final product images, which can be very different. For example, capsules generally evolve into tablets of varying dosage strengths and, perhaps a controlled or sustained release dosage form is developed. To confound things further, it is well known that only a small percentage of R&D projects actually make it through rigid toxicology and clinical programmes to become a successful product. A project can be terminated at any time during development such that it is difficult to justify purchase of a robotic system for any individual project. Finally, a unique attribute of stability studies conducted in analytical R&D laboratories is that samples are not, generally, placed on stability at the same time. They are manufactured, sometimes months apart. In addition, new studies are conceived, very often, as a outgrowth of success in the clinic. New dosage strengths or the potential for a controlled or sustained release dosage form are identified in this fashion. Therefore, workload can not easily be pooled to obtain maximum efficiency of a robotic system. Current robotic technology, however, has greatly enhanced the flexibility of interchanging configuration and method development time has been greatly reduced. Today, it is not unreasonable to conceive of using a single robotic system to facilitate 10–15 projects. Simplified configurations and programming have allowed relatively inexperienced staff to be adequately trained for an analysis in as little as one day. Next to the flexibility of interchanging components, the most significant improvement has been in the introduction of programmes which facilitate mixing of different strengths and dosage forms of a single product. Sample weights and prescribed dilutions can be stored in memory: printouts denote both dosage form and dilution. The ability to correlate 'intended concentration' with appropriate dilutions had allowed samples of a compound to be analysed using the same robotic method employed for defined dosage forms in other matrices, for example for feed anlayses employed in toxicology studies. System productivity software can optimize robotic manipulations when a variety of steps are used. Concurrent software allows 'non-robotic' steps, such as filtration and centrifugation, to proceed independently. Programmes can be developed as canned systems, which allow certain sample manipulations, like liquid/ solid extractions, to be utilized in common, one product or another. Alternatively, one can modify programme steps or string programmes together. All of these relatively recent features have allowed methods to be developed in days not weeks, using all laboratory staff, not a specially trained few. The latter are still needed but can be reserved for more sophisticated problems or for preventative maintenance and calibration.

Method transfer is a stringent requirement for analytical R&D laboratories. The principal goal, once stability has been demonstrated and specifications developed, is to transfer analytical methods, developed in our laboratories, to other potential testing sites which will be required to use them to release new products and continue marketed product stability programmes. Method transfer requires a strong interface with quality control staff. Protocols need to be mutually agreeable. Robotic and manual methods must be provided in addition to validation reports which demonstrate both are accurate and precise and statistically correlate. Procedures must be written, step-by-step, and catalogued in order to track revisions. System suitability requirements must encompass both the manual and robotic versions. Logic diagrams must be provided along with system security. Finally, statistical analysis criteria must allow manual and robotic versions to be compared between laboratories.

To demonstrate the effect these improvements have contributed to enhanced feasibility for robotics in an R&D environment, specific elements of a method development protocol were compared using manual and robotic methods. Development times were tabulated for single component developmental dosage forms. Both capsule and tablet formulations were considered at dosage strengths of 25 mg and 100 mg for both formulations. The analytical method consisted of a liquid/solid extraction, dilution and an isocratic HPLC determination: a 'dilute and shoot method'. A common requirement was the expenditure of 13 days (34% of total effort) to develop and validate the HPLC system, an enantiomeric assay (two centres; four isomers). This time would have been required for either a manual or robotic method. Sample preparation required three days (7% of total effort). Validation of the manual component of the method required 10 days (25% of total effort). Robotic programming and validation required only three days (7%) and six days (15%), respectively. Documenting the methods, exclusive of validation reports required five days (12% of total effort). Total method development time amounted to 40 days for both manual and robotic versions of this method, a reasonable time frame for the manual method *alone*. This same procedure was further evaluated by comparing actual operatator analysis times, manual vs robotic versions. It should be noted that this experiment was designed to include the effective contribution of automated versus manual injections. The data show that, manually, sample preparation of 18 samples (36 determinations) plus two standards required approximately 6·3 h of operator time, compared to just under 1 h employing the robot. Overall, conducting the analysis using robotics to facilitate sample manipulations and autoinjectors reduced the total analysis time from 12·7 h to just under 4 h. Assessments of this type are necessary to demonstate the practical utility of automation for a given analysis. Efficiency may decrease when the number of samples is reduced. However, practical utilization of robotics is obviously dependent on the number of manipulations required in sample preparation. For most dilute and shoot methods, sample analyses requiring in excess of approximately 20–25 injections effects optimum productivity. In this particular example, the operator would have ample time, in day one, to complete final assay reports/notebook entries plus prepare samples for the second day's run. It is important to note that the analysis continues after the chemist has left for the day and, therefore, successful application was limited to a shift operation. It should be apparent that this assessment would have reached very different conclusions, were the laboratory using two shifts (see tables 2 and 3).

To give a very different point of view, the use of automation in dissolution testing will be considered next. Overall, it has been found that the gain in productivity is optimal for large volume samples requiring shorter dissolution run times. Therefore, in the author's laboratories, robotics are best suited to dissolution testing of immediate release dosage forms which require 1 h run times and testing of six individual tablets. Automated testing of controlled or sustained release products has its limitations.

Dissolution testing is normally conducted in either of three modes:

*Table 2. Person-days required for method development.*

Product: single component – capsule and tablet
Dose: 25 mg and 100 mg per unit dose
Principle: liquid/solution extraction; dilute/shoot; HPLC

| Activity | Days required | % total effort |
|---|---|---|
| Development LC system: Enantiometric assay with two centres (four isomers) | 13 | 34% |
| Sample preparation | 3 | 7% |
| Validate manual method | 10 | 25% |
| Robotic programming | 3 | 7% |
| Robotic validation | 6 | 15% |
| Document methods (Not validation reports) | 5 | 12% |
| Total | 40 | 100% |

*Table 3. Comparison of operator times: robotic versus manual for typical stability analysis.*

Assignment: 18 samples plus two standards

| Activity | Manual (h) | Robotic (h) |
|---|---|---|
| Equipment set-up | 0·25 | 0·25 |
| Prep standards | 0·50 | 0·50 |
| Add samples | 0·10 | 0·10 |
| Add solvent | 1·00 | 0·00 |
| Disint/diss samples | 0·50 | 0·00 |
| Add internal standard | 2·00 | 0·00 |
| Sample mixing | 0·50 | 0·00 |
| Centrifugation/setting | 0·10 | 0·00 |
| Aliquoting | 0·10 | 0·00 |
| Dilution/mixing | 0·75 | 0·00 |
| Fill LC vials | 0·50 | 0·00 |
| Subtotal | 6·30 | 0·85 |
| Prepare mobile phase | 1·00 | 1·00 |
| Equilibrate system | 2·00 | 2·00 |
| Injection 51 samples (presumes 4 min chromatography) | 3·40 | 0·00 |
| Total | 12·70 | 3·85 |

(1) *All-manual:* preparation of all solvents; filling of dissolution pots; sampling at desired intervals; spectrophotometric or chromatographic measurements; calculations.

(2) *Semi-automated:* sampling at desired intervals; spectrophotometric or chromatographic measurements; calculations

(3) *Robotic:* all steps exclusive of preparation of dissolution medium.

In a typical 1 h dissolution test of six tablets (immediate release), using four sample intervals, the data show the following:

| Dissolution mode | Number of runs/ day possible | Operator time |
|---|---|---|
| Manual | 2 | 8 h |
| Semi-automated | 3 | 6 h |
| Robotic | 12 | 7.5 h |

Measuring the cost outlay (operator and equipment), the data show the following:

| Dissolution mode | Operator/ equipment cost | Runs/year optimal | Total cost per run |
|---|---|---|---|
| Manual | $64 M | 400 | $160.00 |
| Semi-automated | $92 M | 600 | $153.00 |
| Robotic | $200 M | 2400 | $83.00 |

It can be concluded that 2400 dissolution runs of this type can achieve a saving of $185 000 in one year, assuring rapid payback for the robot.

On the other hand, similar assessments for controlled or sustained release products show that only for an '8 h product' does robotics contribute measurably to productivity. In fact, for '16 h and/or 24 h products', there in no particular cost saving associated with automating dissolution.

### Spreadsheet

All good laboratories develop a calendar year plan. These are used by management to estimate resource utilization for ongoing projects and to project resource needs to meet goals for new projects. However, in R&D, priorities can change rapidly. In addition, new projects can be initiated during the course of a year when potential new drug candidates are identified by the discovery laboratories. Potential licensing candidates are under review constantly, and can, very rapidly, become new projects. At Schering, priorities are reviewed monthly by management. Most often, for the analytical laboratories, a decision to move a project to a lower level of priority has very little impact once stability studies have been initiated because this work can not be interrupted without losing valuable data. As pointed out earlier, adding even small projects has a dramatic impact on analytical testing. As a consequence, unless a project is cancelled, analytical workload tends to increase during the course of a year, not decrease. As a general rule, the larger the analytical department, the more difficult it becomes to credibly justify to management the point at which additional workload can not be absorbed. Whole tracking projects versus headcount utilization remains important, this is effectively accomplished through time cards and project codes. However, this is mostly 'retrospective' and does not really capture the magnitude of analytical testing which goes into a project. We have found that an independent spreadsheet analysis, which categorizes and counts the various kinds of analyses which are carried out, presents a very credible overview of headcount utilization and needs for management.

There is abundant availability of spreadsheet software packages. Technology exists for computer-assisted interfacing between stability protocols and assay requests using a Laboratory Information Management System (LIMS). However, the author's laboratory uses an independent spreadsheet system. Laboratory managers provide all input data which helps them to remain close to those day-to-day operations. First, managers categorize all analytical determinations carried out in their respective laboratories on a monthly basis. Many, if not most, chemists independently summarize their activities daily: the system simply requires they keep a written record of this, with the total number of determinations, by category, provided to their managers on a monthly basis. Fourteen categories are utilized but, generally, a chemist does not exceed one or two catagories on any given day. Total determinations, by laboratory/category, are incorporated into spreadsheet formulas which then show relationships between monthly/yearly available work days and the number of available chemists. The data are corrected for laboratory outages due to illness or openings. For example the data show that, for the first eight months in 1992, the author's laboratories generated approximately 162 000 determinations, averaging 953 determinations per working day and 10·9 determinations per bench chemist per day. The spreadsheet presents simple graphical summaries of workload distribution, by category and laboratory, for management review. For example, in this period, the data show that 20% of laboratory efforts went into method development; 8·6% to releasing clinical supplies; 3·8% to certifying reference standards; 5% to instrument calibration and 16% to screening 'candidate' formulations. Summaries can be focused on individual laboratories or the entire department. With few exceptions, the laboratories are organized such that different laboratories become involved as a project progresses through clinical development. Therefore, it can be identified quickly if more resources are necessary because 'new chemical entities' are entering development, as opposed to those projects which are consuming resources related to: testing to support proposed 'line extensions', technical support to manufacturing etc. The system is very useful in documenting the utilization of resources and, overall, maintaining the credibility of the analytical laboratory (see tables 4 and 5).

*Table 4. 1992 workload summary.*

| Source | Jan | Feb | Mar | ... | Total |
|---|---|---|---|---|---|
| PSRs | 1 228 | 899 | 1 298 | ... | 7 166 |
| Methods development | 3 344 | 3 688 | 3 909 | ... | 32 686 |
| PMRs | 1 085 | 549 | 521 | ... | 6 592 |
| Instrument calibration | 1 328 | 1 256 | 1 028 | ... | 8 909 |
| Miscellaneous | 1 630 | 594 | 1 255 | ... | 9 473 |
| PRNs | 9 011 | 7 660 | 8 421 | ... | 52 842 |
| QC support | 105 | 57 | 233 | ... | 1 876 |
| Raw material | 855 | 1 263 | 1 275 | ... | 5 844 |
| Special PRNs | 2 361 | 2 778 | 2 681 | ... | 26 367 |
| Toxicology support | 249 | 342 | 284 | ... | 1 638 |
| Special services | 109 | 57 | 129 | ... | 827 |
| Standards | 829 | 1 310 | 968 | ... | 6 362 |
| Contract laboratory data | 1 108 | 408 | 276 | ... | 3 599 |
| Cleaning validations | 150 | 79 | 160 | ... | 1 376 |
| Total | 22 284 | 208 922 | 22 152 | ... | 161 958 |

* Contract labatory *not* included

*Table 5. 1992 Workload summary.*

| | Jan | Feb | Mar | ... | Total |
|---|---|---|---|---|---|
| Available work days | 22 | 19 | 22 | ... | 170 |
| Total determinations | 22 284 | 20 892 | 22 152 | ... | 161 958 |
| Determinations/day | 1 013 | 1 100 | 1 007 | ... | 953 |
| Total chemists | 88·8 | 88·8 | 89·8 | ... | 87·6 |
| Determinations per man day | 11·41 | 12·93 | 11·22 | ... | 10·88 |

### Summary

Automation in the analytical R&D laboratory can be productive with tangible payback. While certainly optimal, it is not necessary to fully integrate various systems to achieve this. In fact, there is a certain vulnerability associated with system integration although I believe developments' future will minimize concerns in this regard. Computers have been the principal factor in all of these accomplishments. While this discussion has been limited to only a few applications these examples demonstrate the importance of assessing individual laboratory needs carefully before making capital and resource commitments to automation. This should include a plan to capture 'payback versus payout' once the systems are in place.

### Acknowledgements

The author gratefully acknowledges the help of Scott Blumenreich, MS Group Leader, PACRD, Computer Sciences Ed Mularz, PhD., Group Leader, PACRD, Dosage Form Analysis Jack Rosen, MS, Associate Director, Dissolution/Novel Dosage Form Analysis.

